# Bioprospecting of novel silica solubilizing bacteria as bioinoculants for sustainable silica management

**DOI:** 10.3389/fmicb.2025.1556406

**Published:** 2025-06-09

**Authors:** Elina Maharjan, Sonam Mahawar, Surya Chauhan, Sudhir Kumar Upadhyay, Santosh Ranjan Mohanty, Ajaz Ahmad, Rajesh Kumar Singh, Devendra Jain

**Affiliations:** ^1^Central Department of Microbiology, Tribhuvan University, Kirtipur, Nepal, India; ^2^All India Network Project on Soil Biodiversity- Biofertilizers, Department of Molecular Biology and Biotechnology, Maharana Pratap University of Agriculture and Technology, Udaipur, India; ^3^Research and Development Cell, Lovely Professional University, Phagwara, Punjab, India; ^4^Indian Institute of Soil Science, Indian Council of Agricultural Research, Bhopal, Madhya Pradesh, India; ^5^Department of Clinical Pharmacy, College of Pharmacy, King Saud University, Riyadh, Saudi Arabia; ^6^Key Laboratory of Sugarcane Biotechnology and Genetic Improvement (Guangxi), Ministry of Agriculture, Sugarcane Research Center, Chinese Academy of Agricultural Sciences, Nanning, Guangxi, China

**Keywords:** silica solubilizing rhizobacteria, mineralization, phyto-stimulation, antioxidants, ARDRA, 16S rDNA

## Abstract

Silicon (Si) is important quasi-essential element, important for growth and productivity in plants by abetting abiotic and biotic stresses. In the recent times intensive cultivation in India has led to depletion of available Si in soils leads stagnation in the crop productivity. In this study, out of 88 rhizobacterial isolates, 24 potential isolates having significant silica solubilizing capability and exhibited plant growth-promoting characteristics were characterized at biochemical and molecular level and further to study their effect on plant growth stimulation and augment the absorption and accumulation of active silica in plants. In qualitative method, all 24 SiS-RB isolates were able to form clear zone of silica solubilization with the solubilizing index (SSI) in the range of 1.05–3.40 cm, whereas in quantitative silica solubilization the solubilized silica was observed in a range of 1.29–43.29 ppm. The 24 SiS-RB isolates further demonstrated plant growth promoting activities. Subsequently, these isolates were evaluated for their capacity to solubilize various minerals, including biotite, calc silicate, feldspar, muscovite, orthoclase, and quartzite, revealing that only six isolates had significant solubilization ability. The six potent isolates *viz.* SSB-2, SSB-8, SSB-11, SSB-12, SSB-21, and SSB-24 showed a considerable enhancement in maize plant development under *in vitro* conditions, including improved antioxidant properties such as catalase (CAT), superoxide dismutase (SOD), peroxidase (POD), polyphenol oxidase (PPO), and phenylalanine ammonia lyase (PAL) activities. All 24 SiS-RB were subsequently analyzed for genetic diversity using amplified ribosomal DNA restriction analysis (ARDRA) analysis, and findings revealed that considerable higher genetic diversity exists among SiS-RB isolates. The integrated dendrogram exhibited similarity indices between 0.11 and 0.90, with a mean of 0.51. All potent silica-solubilizing plant growth-promoting rhizobacterial isolates were identified using 16S rDNA sequencing and belongs to *Enterobacter* sp., *Serratia surfactantfaciens*, and *Klebsiella* sp. These influential isolates would significantly enhance silicate management through Si based biofertilizer development for plant growth promotion under Si deficient soils.

## 1 Introduction

Silicon (Si) is considered a “quasi-essential” or advantageous element, and it has a notable function as a micronutrient for plants (Pavlovic et al., 2021; Thakral et al., 2024). Silicon is primarily found on Earth in numerous forms, including wollastonite, feldspar, Si dioxide, quartz (pure SiO_2_), and other clay minerals such as kaolinite, mica, and silicates that include elements like aluminum, magnesium, calcium, salt, potassium, or iron (Tayade et al., 2022; Thakral et al., 2024). The use of Si in agricultural fertilization has become more popular due to its non-corrosive nature and sustainability (Thakral et al., 2024).

Plants assimilate Si from the soil as monosilicic acid (H_4_SiO_4_), which is then transported throughout the plant’s tissues, primarily the cell wall and epidermis (Zexer et al., 2023). This integration boosts the rigidity of the cell wall, promotes the organization of the leaf, and may increase the ability to perform photosynthesis in some species (Zexer et al., 2023). Moreover, Si has a crucial function in augmenting plant immunity against diseases and pests, minimizing water loss via transpiration, and improving water use efficiency (Yang et al., 2022). The presence and dispersion of Si in soils are affected by variables such as soil origin, climate, texture, and the extent of soil erosion. Severe weathering may result in the depletion of Si from soils, resulting in the formation of fewer plant-accessible compounds (Katz et al., 2021). In addition, contemporary agricultural methods such as widespread farming and the use of phytosanitary substances and NPK fertilizers might exacerbate the decline of Si levels in soil (Kovács et al., 2022).

Silicon plays a multifaceted role in plant defense, offering protection against viruses, fungi, and herbivores at various growth stages. It acts by repelling pests, blocking their penetration, and lowering the harm they cause. Si not only creates physical barriers, but also plays a role in stimulating systemic resistance in plants (Singh et al., 2020). This process involves the activation of defense-related enzymes such as peroxidase (POD), polyphenol oxidase (PPO), and phenylalanine ammonia-lyase (PAL). These enzymes have essential functions in the plant’s reaction to pathogen invasion, such as the production of lignin and the formation of phenolic compounds via the phenylpropanoid pathway (Alhousari and Greger, 2018; Singh et al., 2020).

Silicon exists in soils in both amorphous and crystalline states, and may be found in minerals such as kaolin, smectite, vermiculite, and quartz. While Si makes up a considerable part of the Earth’s crust, it is usually insoluble, which limits the amount of Si that plants can absorb (Etesami and Maheshwari, 2018). Silicon is released by weathering processes or the actions of soil microbes and plants, which breakdown it into soil water. Nevertheless, soils in tropical climates, which are known for their extensive weathering, sometimes suffer from a scarcity of plant-accessible Si (Khan, 2025). Continuous cultivation of Si-demanding crops may result in significant depletion of soil Si, especially in sandy soils used for crops such as sugarcane. Plant tissues, including as husks, leaves, and stems, are the main sites of Si accumulation (Katz et al., 2021). The advantages of this extend to mitigating the impact of both biotic stressors, such as diseases and pests, and abiotic stressors, such as drought, salt, and heavy metal toxicity. Enhancing the absorption of Si in crops is seen as a sustainable approach to improve production under challenging environments. Silicon enhances the structural integrity of plant cell walls, hence enhancing the ability of crops such as rice, barley, wheat, and cucumbers to withstand different types of stress (Zargar et al., 2019). On the other hand, a lack of Si makes plants more susceptible to assaults from pests and pathogens (Islam et al., 2020).

Microorganisms, namely bacteria from the genera *Bacillus*, *Pseudomonas*, and *Burkholderia*, have the potential to dissolve silica and silicate minerals, which in turn increases the availability of Si for plants (Bist et al., 2020). Hence, the presence of silica-solubilizing bacteria (SiS-B) can enhance plant health, soil fertility, and defense systems. Biofertilizers containing SiS-B are becoming increasingly recognized as a sustainable and eco-friendly substitute for traditional Si-fertilizers, which may pose environmental risks and lead to higher production expenses. Silica-solubilizing-bacteria based biofertilizers transform insoluble silicates in the soil into soluble forms that plants may easily take up, providing a cost-efficient method to enhance Si accessibility and agricultural output. Rhizospheric bacteria have silica-solubilizing abilities and may produces phytostimulants, biocontrol agents, and other compounds that promote development in plants, which are together, termed silica-solubilizing plant growth-promoting rhizobacteria (SiS-PGPR) (Chaganti et al., 2023). SiS-PGPR significantly contribute to the mineralization of silica from sequestered silica in the soil (Etesami and Schaller, 2023). The use of local SiS-RB isolates specific to geographic locations will provide the advantage of quick adaptation and less competition when introduced in rhizosphere will make them ideal choice to mitigate environmental stresses. Consequently, this research aimed to (i) isolate and screen silica solubilizing rhizobacteria (SiS-RB), (ii) examine additional characteristics such as mineral solubilization and plant growth-promoting attributes, (iii) investigate the impact of effective SiS-PGPR on maize plant growth performance, and (iv) conduct molecular diversity and identification of potent SiS-PGPR.

## 2 Materials and methods

### 2.1 Sample collection and rhizobacterial isolation

Soil samples from the rhizosphere of maize were collected from different sites of Kumbhalgarh district. At each site, 100 g of root-adhering soil were carefully collected in sterile plastic bag and stored at 4^^°^C in lab. For the isolation of rhizobacteria, root-adhering soil was serially diluted and 10^–4^ and 10^–6^ dilution was used for rhizobacteria isolation by using Nutrient Agar, King’s B, and Jensen N-Free media to obtained morphologically different rhizobacteria as previously outlined by Upadhyay et al. (2009).

### 2.2 Silica, other mineral solubilizing attributes, including phyto-stimulation analysis

Qualitative investigation of silica solubilization was conducted using 2.5 μl of pre-incubated bacterial culture disseminated over Bunt and Rovira agar supplemented with 0.25% magnesium trisilicate (w/v). The plates were incubated in darkness at 28 ± 2°C for 72 h, following which a clear zone around the bacterial colony was detected (Bunt and Rovira, 1955). The solubilizing capability was evaluated using the Solubilizing Index, computed as the ratio of the overall diameter of the colony. Quantitative silica solubilizing activities were assessed using 100 ml of bacterial culture cultivated in Bunt and Rovira broth, supplemented with 0.25% magnesium trisilicate (w/v), for 7 days, followed by centrifugation at 10,000 rpm for 15 min. One milliliter of the supernatant was combined with reagents and evaluated with the silicic acid-molybdate technique as described by Santi and Goenadi (2017).

Various minerals, including Biotite, Calc-silicate, Feldspar, Muscovite, Orthoclase, and Quartzite, were used to assess the solubilization capability of bacteria. The silica solubilization concentration of various SiS-B isolates was measured after 5 and 10 days. Phosphate solubilization was assessed using 2.5 μl of pre-incubated bacterial culture disseminated over Pikovskaya’s agar enriched with 0.5% calcium phosphate. A transparent halo zone around the bacterial colony was noticed after 24 h (Pikovskaya, 1948). Zinc solubilization was evaluated by Krithika and Balachandar (2016), potassium solubilization was analyzed by method outlined (Saheewala et al., 2023), and siderophore production was measured using the method outlined by Schwyn and Neilands (1987). The biochemical tests, including catalase test, gelatin liquefaction, starch hydrolysis, oxidase test, and citrate utilization, were assessed using conventional protocols. The synthesis of phytohormones, namely IAA and Gibberellic Acid, was assessed using the methodologies of Gordon and Weber (1951), Berríos et al. (2004), respectively. The formation of hydrogen cyanide and ammonia, together with ACC deaminase activity, was evaluated in research conducted by Bakker and Schipper (1987), Penrose and Glick (2003), respectively.

### 2.3 Pot experiments

The pot studies were conducted under net house conditions using a complete random design (CRD) with triplicates for each treatment, outlined by Sukhwal et al. (2023), Upadhyay and Chauhan (2022). Each pot included 250 g of dirt from the Kumbhalgarh district (25°8′56″N 72°34′49″E), Rajasthan. The seeds of the cultivable maize variety were surface sterilized and inoculated on a 0.8% agar plate to facilitate germination, as outlined by Saheewala et al. (2023). Following germination, seeds of comparable size were transferred into pre-treated pots. Each pot containing germinated seeds received 2 ml of silica-solubilizing rhizobacterial culture, which had been pre-incubated for 24 h at 38 ± 2°C, as per Saheewala et al. (2023). The treatments T1 = SSB2, T2 = SSB8, T3 = SSB11, T4 = SSB12, T5 = SSB21, and T6 = SSB24, together with a control group (without SiS-PGPR inoculation), were formulated using effective SiS-plant growth-promoting rhizobacteria. Plant growth metrics, such as root and shoot fresh weight, shoot length, root length, and chlorophyll content, were assessed after 14 and 28 days, respectively. The chlorophyll-a content was quantified using a UV-visible spectrophotometer (Arnon, 1949). Furthermore, stress-related enzymes including Catalase (CAT), Superoxide Dismutase (SOD), Peroxidase (POD), Polyphenol Oxidase (PPO), and Phenylalanine Ammonia Lyase (PAL) were evaluated by methodologies delineated in our prior publication (Upadhyay et al., 2012; Jain et al., 2017).

### 2.4 Genetic fingerprinting, Molecular characterization, and phylogenetic analysis

The total genomic DNA of SiS-RB was extracted using the GenElute Bacterial Genomic DNA Kit (Sigma, United States). The 16S rDNA region was amplified using the universal primers (Forward 5′-AGAGTTTGATCCTGGCTAG-3′and Reverse 5′-AGGAGGTGATCCAGCCGCA-3′) (Edwards et al., 1989). To assess genetic similarity, ARDRA (Amplified Ribosomal DNA Restriction Analysis) banding patterns were analyzed using Jaccard’s coefficient. The resulting similarity coefficient matrix is then processed using the Unweighted Pair Group Method with Arithmetic Mean (UPGMA) algorithm to generate clusters. This analysis is performed using the NTYSYS 2.02 PC program. The amplified 16S rDNA products were then subjected to restriction digestion with endonucleases such as *Alu*I*, Hae*III*, Hin*fI, and *Taq*I. PCR amplified 16S rDNA was purified using a QIA-PCR purification kit (Qiagen) and sequenced on an Applied Biosystems (ABI) prism automated DNA sequencer (3,130 × 1). The obtained nucleotide sequences were aligned with the GenBank using NCBI BLAST, and the partial 16S rDNA sequences were submitted to NCBI GenBank. Genomic sequences of rhizobacterial isolates were subjected to molecular evolutionary studies, and a phylogenetic tree was constructed using BEAST software.

### 2.5 Statistical analysis

The study used triplicate experimental data for reliability and repeatability, and analyzed statistically using SPSS and OriginPro software. A dendrogram was created using NTYSYS software to understand linkages and evolutionary patterns. A phylogenetic analysis was conducted using the online BLAST program, which offers advanced computational techniques for Bayesian phylogenetic inference.

## 3 Result and discussion

### 3.1 Isolation of silica solubilizing bacteria from various sources

The determination of maximal microbial diversity in the rhizospheric zone used several mediums, as previously reported by multiple studies. Various media possess distinct nutrition sources, allowing microbes to proliferate according to their specific nutrient and energy requirements (Upadhyay et al., 2022). This work used several mediums for the isolation of silica-solubilizing rhizobacteria (SiS-RB). Nutrient agar enriched with magnesium trisilicate (Mg_2_O_8_Si_3_, ∼0.25%) has been used to distinguish silica-solubilizing bacteria from other bacterial species, as shown by Vasanthi et al. (2018). Researchers have often used Modified Bunt and Rovira media containing magnesium trisilicate for the isolation of silica-solubilizing bacteria (Chandrakala et al., 2019). The dominant technique is magnesium trisilicate, which enables the detection of SiSB by the creation of a distinct solubilization zone around bacterial colonies. This research further screened silica-solubilizing rhizobacteria for growth using a modified Bunt and Rovira medium enriched with magnesium trisilicate. Of the 88 rhizobacterial isolates derived from various media, only 24 shown the ability to solubilize silica on Bunt and Rovira medium, and these 24 silica-solubilizing rhizobacterial isolates were subsequently used in this investigation. Similarly, Cruz et al. (2022) identified 130 bacterial strains from field-cultivated sugarcane, rice, wheat, maize, and soybean in diverse locales around Louisiana. Among them, 20 strains were classified as silica-solubilizing bacteria, using various media such as Luria broth (LB) agar, tryptic soy agar (TSA), and silica broth and agar medium.

### 3.2 Morphological characterization of silica solubilizing bacteria

All 24 screened silica solubilizing rhizobacterial isolates were exposed to morphological characterization for further investigation and results were summarized as shown in Supplementary Table 1. Gram staining is a most useful and important tool to differentiate bacteria based on wall composition, it’s helpful beyond the genus level by providing both biochemical information about the composition of bacteria and special information about the distribution of chemicals into the wall (Beveridge, 2001). Morphological characteristics of the colony were recorded by gram staining which revealed SiSB as mostly gram negative except SSB-18 and SSB-23. Majority of the SiS-RB isolates were rod in shape with few isolates that are coccoid (Table 1). Similarly, Sulizah et al. (2018) characterized five isolates of silica solubilizing bacteria in terms of morphological characterization, and reported that all the isolates were gram negative.

**TABLE 1:** Quantitatively and qualitatively estimation of silica solubilization attributes of silica solubilizing rhizobacteria (SiS-RB) isolates.

SiS-RB isolate	Solubilization index (cm)	*In vitro* silica solubilization (ppm)	Gram staining*	Shape
SSB-1	1.87 ± 0.26	3.12 ± 0.9	Gm-ve	Cocci
SSB-2	2.6 ± 0.3	18.32 ± 2.1	Gm-ve	Cocci
SSB-3	1.14 ± 0.36	18.14 ± 2.1	Gm-ve	Cocci
SSB-4	1.22 ± 0.31	8.54 ± 1.3	Gm-ve	Cocci
SSB-5	1.076 ± 0.025	5.69 ± 1.2	Gm-ve	Cocci
SSB-6	2.09 ± 0.01	1.29 ± 0.6	Gm-ve	Cocci
SSB-7	1.09 ± 0.15	4.83 ± 1.1	Gm-ve	Cocci
SSB-8	3.4 ± 0.15	15.97 ± 2.3	Gm-ve	Cocci
SSB-9	1.42 ± 0.20	7.691 ± 1.2	Gm-ve	Cocci
SSB-10	1.1 ± 0.25	12.03 ± 1.6	Gm-ve	Cocci
SSB-11	2.66 ± 0.25	15.74 ± 2.3	Gm-ve	Cocci
SSB-12	2.85 ± 0.030	15.63 ± 2.5	Gm-ve	Cocci
SSB-13	1.05 ± 0.079	11.17 ± 1.6	Gm-ve	Rod
SSB-14	1.09 ± 0.04	17.63 ± 2.1	Gm-ve	Rod
SSB-15	1.09 ± 0.01	4.32 ± 1.1	Gm-ve	Rod
SSB-16	1.2 ± 0.173	14.83 ± 1.5	Gm-ve	Rod
SSB-17	2.16 ± 0.25	4.32 ± 0.5	Gm-ve	Rod
SSB-18	1.1 ± 0.15	12.66 ± 1.3	Gm+ve	Rod
SSB-19	1.14 ± 0.020	6.6 ± 0.6	Gm-ve	Cocci
SSB-20	2.01 ± 0.15	10.26 ± 1.9	Gm-ve	Rod
SSB-21	2.4 ± 0.2	18.2 ± 2.6	Gm-ve	Rod
SSB-22	1.076 ± 0.25	4.66 ± 1.22	Gm-ve	Rod
SSB-23	1.066 ± 0.03	5.97 ± 1.2	Gm+ve	Cocci
SSB-24	2.8 ± 0.25	43.92 ± 4.35	Gm-ve	Rod

*Gm+ve, Gram positive; Gm-ve, Gram negetive.

### 3.3 Qualitative and quantitative analysis

All 24 SiSB isolates were able to form clear zone or halo zone of silica solubilization on Bunt and Rovira Agar plate supplemented with silica salt. Silica solubilization was measured as Solubilizing Index (SI) which ranged from 1.05 to 3.40 cm as shown in Table 1. Among 24 isolates, SI was maximum recorded for SSB-8 (3.4 ± 0.15) followed by SSB-24 (2.8 ± 0.25) whereas the minimum SI was observed in SSB-13 (1.05 ± 0.079). Babu et al. (2022) reported SI of silica solubilizing bacterial isolates ranging from 2.64 to 4.95. Chopra et al. (2021) reported SI of six solubilizing bacterial isolates were ranging from 1.09 to 2.66. Among them, SSB-24 exhibited the highest solubilization, with 43.92 ppm, while SSB-2 achieved 18.32 ppm, and SSB-6 recorded the lowest at 1.29 ppm. Similar findings were reported by Babu et al. (2022), where SiKPP-1 demonstrated the highest silica content of 2.16 ppm, followed by SiPYY-3 at 2.12 ppm, and SiAGG-1 with 0.52 ppm. Sulizah et al. (2018) reported that OS12 had the highest silicate solubilization of 1.053 ppm in Bunt and Rovira broth.

**FIGURE 1:**
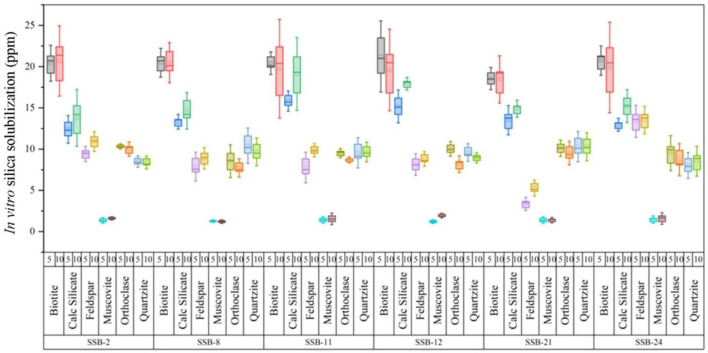
*In vitro* silica solubilization ability of potent rhizobacterial isolates against different silicate minerals after 5 and 10 days, respectively. Error bar reparents standard deviation.

Variation between qualitative and quantitative screening methods was observed, indicating differences in solubilization of inorganic silicates between plate and liquid assays. Isolates that demonstrated high solubilization index (SI) on solid media did not necessarily exhibit high dissolution in liquid assays reported by Vasanthi et al. (2018). Out of 24, six SiS-RB isolates were solubilized highest silica content in biotite followed by Calc-silicate and feldspar (Figure 1). SSB-8, SSB11, SSB24 were highest solubilizing ability of biotite mineral (Figure 1). Muscovite mineral was least solubilized by all the isolates. Our results were similar to Vasanthi et al. (2018) proportion of SiSB associated with different minerals does not directly correlate with the silica content of the minerals. For instance, muscovite, which contains 21% silica, harbored a higher proportion of SSB compared to phyto-sil, which has 78% silica. In contrast, quartz, with 98% silica, talc with 54%, and feldspar with 45% silica, exhibited lower proportions of SiSB. These findings highlight a significant discrepancy between the total bacterial populations found in soil or silicate minerals and the specific SiSB isolates.

Silica-solubilizing microorganisms have the capability to release soluble silica from insoluble inorganic silicates (such as those containing calcium, aluminum, potassium, and magnesium) and biogenic materials like diatomaceous earth, siliceous earth, rice husk, and rice straw. The formation of a halo zone in agar media can be influenced by several factors, including the type of substrate, the size and volume of the inoculant, the thickness of the agar layer, medium composition, pH, and incubation temperature (Chaganti et al., 2023). Organic acids produced by silica-solubilizing bacteria are key to breaking the Si-oxygen bonds (O-Si-O) in quartz, thereby releasing soluble silica (Etesami and Glick, 2023). In this study, organic acids were analyzed by using HPLC (Data are not shown). These acids, such as acetic acid and citric acid, play a crucial role in the dissolution process by producing hydrogen ions (H^+^), which can help dissolve silicate minerals (Figure 1). Organic acids, particularly those containing carboxylate groups, are weak acids that easily ionize, facilitating the release of cations and affecting the pH during silica solubilization (Chaganti et al., 2023). An excess of cations can influence the pH and pull anions like hydroxide (OH^–^) away from quartz, aiding in its dissolution. The chelation of anions by organic acids results in the solubilization of quartz into a form that can be absorbed by plants, specifically as monosilicic acid [Si(OH)_4_], which is taken up by paddy plants through their lateral roots.

### 3.4 Plant growth promoting activities of potent SSB

All SiS-RB isolates were evaluated for their plant growth-promoting attributes as shown in Supplementary Tables 2, 3. These activities positively impact on plant growth by producing growth regulators, enhancing et al., 2022). nutrient availability, and protecting plants from various abiotic and biotic stresses (Upadhyay In addition to effective silica solubilization and plant growth promoting attributes of SiS-RB, increases their applicability in silica management and plant growth performance, therefore this study plays remarkable role in silica management for plant growth.

Indole 3-acetic acid (IAA) has been reported to play a key role in plant growth promotion (Shoebitz et al., 2009; Upadhyay et al., 2022) and play important role in biotic and abiotic stresses. It is well-known that auxin as important phytohormone enhances the plant growth and development from seedling stage to senescence (Mazzoni-Putman et al., 2021). It is well-known that the presence of auxin enhances the influence of bacteria in the rhizosphere of a plant. All the SiS-RB isolates were IAA producer as sown in (Supplementary Tables 2, 3). Cruz et al. (2022) demonstrated that all silica solubilizing bacterial isolates produced indole IAA with the range of 1.97–77.32 μg/ml. All the six SiS-RB isolates produce GA_3_ which ranges from 0.378 to 0.705 μg/ml. SSB-24 produces highest amount of GA_3_ (0.711) followed by SSB-2 (0.705) and SSB-12 (0.652) and SSB 8 (0.645) whereas lowest in SSB 22 (0.378) followed by SSB 23 (0.387) (Supplementary Tables 2, 3). Similarly, Upadhyay et al. (2009) investigated the efficiency of salt-tolerant rhizobacteria for gibberellic acid production. Gibberellic acid is a plant hormone and play remarkable role in plant growth (Upadhyay et al., 2022).

Microorganisms enhance the availability of inorganic phosphorus (P) through the production of protons and organic acids, which are commonly found among rhizosphere P-solubilizing microorganisms (Hinsinger et al., 2011). Out of 24 SiS-RB isolates, 21 isolates were found positive for phosphate solubilization. Among 24 isolates, SSB-24 (3.0 ± 0.21) was shown maximum ability for phosphate solubilization followed by SSB-2 (2.5 ± 0.19) (Table 2). Similarly, Cruz et al. (2022) reported that nine out of twenty silica solubilizing bacterial isolates were able to solubilize tricalcium phosphate in Pikovskaya’s medium, as evidenced by the formation of a clearing zone around the bacterial colonies. Table 2 shows the potassium solublization ability of six SiS-RB only. Potassium is one of the most important macro-nutrients for plants it plays a significant role in enzyme activation, charge balance, osmoregulation and reduction in the negative effects of stress. Among 24 SiS-RB isolates only 22 were found positive for potassium solubilization, SSB-24 (6 ± 0.27) followed by SSB-8 (3.5 ± 0.26) whereas minimum solubilization was recorded in SSB-16 (1.2 ± 0.058). Zinc is an essential micronutrient necessary in trace amounts for optimal growth, reproduction, and cellular metabolism (Vidyashree and Arthanari, 2021). However, elevated levels of zinc in soils can pose environmental hazards and threaten sustainable and high-quality food production. The use of zinc-tolerant microorganisms can help mitigate these issues and manage excessive zinc concentrations (Rani et al., 2023). All SiS-RB isolates were found positive for zinc solubilization as shown in Supplementary Tables 2, 3. The maximum solubilization was recorded in SSB-2 (5 ± 0.33) followed by SSB12 (5 ± 0.21) exhibiting the maximum SI. The SSB 20 (1.42 ± 0.12) found to be least solubilizer (Table 2).

**TABLE 2:** Plant growth promoting attributes, biochemical characteristic, and GenBank-accession number of potent silica solubilizing rhizobacteria (SiS-RB) isolates.

SSB isolate	IAA	GA_3_	Phosphorus solubili-zation	Potassium solubili-zation	Zinc solubili-zation	ACC	Ammonia	HCN	Starch Hydro-lysis	Citrate Utili-zation	Nitrate Reduc-tion	Gelatin lique-faction	Catalase Activity	Oxidase	GenBank-accession number
SSB-2	1.29 ± 0.3	0.71 ± 0.1	2.5 ± 0.19	3 ± 0.25	5 ± 0.33	+	+	+	**+**	**+**	−	−	**+**	−	PQ157604
SSB-8	8.54 ± 1.2	0.65 ± 0.3	1.6 ± 0.198	3.5 ± 0.26	4.2 ± 0.10	++	−	−	**+**	**+**	**+**	−	**+**	**+**	PQ157605
SSB-11	14.83 ± 2	0.62 ± 0.3	1.6 ± 0.18	3.4 ± 0.20	4.25 ± 0.22	+	−	−	**+**	**+**	**+**	−	**+**	−	PQ157606
SSB-12	4.32 ± 1.1	0.65 ± 0.2	1.16 ± 0.22	3 ± 0.179	5 ± 0.21	+	+	+	**+**	**+**	**+**	**+**	**+**	**+**	MW308551
SSB-21	5.69 ± 1.3	0.61 ± 0.2	1.75 ± 0.21	3.25 ± 0.12	3.75 ± 0.27	+	+	+	−	**+**	**+**	−	**+**	−	PQ157607
SSB-24	4.32 ± 1.2	0.71 ± 0.2	3.0 ± 0.21	6 ± 0.27	4.25 ± 0.29	+	+	+	**+**	**+**	**+**	−	**+**	−	PQ157608

Data of only potent silica solubilizing plant growth promoting rhizobacteria (SiS-RB).

Stress can lead to increased production of 1-aminocyclopropane-1-carboxylic acid (ACC), which serves as a precursor to ethylene (Wang and Adams, 1982). It is widely recognized that ACC can be hydrolyzed by the bacterial enzyme ACC deaminase into ammonia and α-ketobutyrate (Sarapat et al., 2020). By reducing the amount of ACC in plants, PGPRs with ACC deaminase activity can promote the development of a more robust root system. In this study, 17 out of 24 SSB strains were found to be positive for ACC deaminase activity, while 7 were negative (Supplementary Tables 2, 3). Similarly, Cruz et al. (2022) reported that 9 out of 20 silica solubilizing bacteria isolates exhibited ACC deaminase activity. Ammonia and HCN production by plant growth-promoting bacteria (PGPB) plays a crucial role in nitrogen supply to host plants, enhancing root and shoot elongation and overall biomass. The ammonia produced by these microorganisms in the soil serves as a valuable nitrogen source for plants. In this study, Table 2 showed the Ammonia and HCN activities by SiS-RB.

### 3.5 *In vitro* efficacy of SiS-RB in maize

**FIGURE 2:**
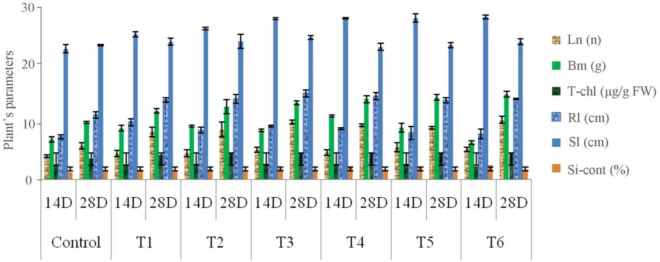
Influence of different treatment of silica solubilizing plant growth promoting rhizobacteria on maize plant growth performance after 14 and 28 days. The error bar represents ± Standard deviation values; (T1 = SSB2, T2 = SSB8, T3 = SSB11, T4 = SSB12, T5 = SSB21, and T6 = SSB24), Sl, shoot length; Rl, root length; Ln, leaf number; Bm, biomass (g); T-chl, total chlorophyll content; Si-cont, Silica content in plant.

*In vitro* studies of silica solubilizing bacteria in maize plant growth-performance were described in Figure 2. The inoculation of silica solubilizing plant growth promoting rhizobacteria significantly increased shoot length of maize T5 (7.56 ± 1.28). Average biomass recorded in maize were found to be maximum in T1 (14.33 ± 2.08) compared to control. With the increase of chlorophyll highest silica content was found in T6 (3.68 ± 0.056) compared to control.

Vandevivere et al. (1994) highlight the role of Si-solubilizing bacteria in consistently providing Si, which contributes to enhanced plant growth characteristics. This is due to their ability to release soluble silica, which can be utilized by plants for improved development and stress resistance. The expression of stress related enzymes viz., Catalase (CAT), Superoxide dismutase (SOD), Peroxidase (POD), Polyphenol oxidase (PPO) and Phenylalanine ammonia lyase (PAL) were also studied in 28 days old maize plantlet and it was significantly influenced by SiS-RB treatment (Table 3). The higher expression of these antioxidant/defense enzyme activities in the maize will not only mitigate stress but also contribute to plant growth.

**TABLE 3:** *In vitro* seed bacterization studies of silica solubilizing plant growth promoting rhizobacteria on stress related enzymes of maize after 28 days.

Treatment	SOD μmol min^–1^ g^–1^	POD μmol min^–1^ g^–1^	PPO μmol min^–1^ g^–1^	PAL μmol min^–1^ g^–1^	CAT μmol min^–1^ g^–1^
Control	9.35 ± 0.61^a^	0.0501 ± 0.0017^a^	0.09 ± 0.014^a^	0.214 ± 0.04^a^	89 ± 9.46^a^
T1	10.09 ± 0.23^b^	0.0833 ± 0.0061^b^	0.12 ± 0.012^b^	0.248 ± 0.03^b^	101.5 ± 3.25^b^
T2	10.64 ± 0.38^b^	0.0851 ± 0.0093^b^	0.15 ± 0.018^c^	0.251 ± 0.07^b^	119 ± 7.39^b^
T3	11.06 ± 0.60^c^	0.0847 ± 0.0102^b^	0.17 ± 0.011^c^	0.268 ± 0.04^b^	120.75 ± 9.87^b^
T4	11.10 ± 1.04^c^	0.0889 ± 0.0122^b^	0.25 ± 0.021^d^	0.280 ± 0.09^b^	155.25 ± 12.05
T5	11.20 ± 0.81^c^	0.0987 ± 0.0142^bc^	0.28 ± 0.025^d^	0.299 ± 0.05^c^	289 ± 9.1^cd^
T6	11.56 ± 0.61^c^	0.0991 ± 0.0152^c^	0.29 ± 0.021^d^	0.287 ± 0.06^b^	300.5 ± 11.54^cd^

Data was collected from triplicate study, DMRT at *p* < 0.05 shows similar letter represent data not significant, while different letters show significant data. T1 = SSB2, T2 = SSB8, T3 = SSB11, T4 = SSB12, T5 = SSB21, and T6 = SSB24.

### 3.6 Molecular characterization using ARDRA

Genetic fingerprinting, a key molecular technique, creates a unique profile of microbial communities through the direct analysis of PCR products from individual strain DNA. This process generates a fingerprint based on either sequence polymorphism or length polymorphism. Genetic fingerprinting is both rapid and capable of analyzing multiple samples simultaneously. While it is effective in demonstrating differences or effects on microbial communities, it does not provide direct taxonomic identities (Lendvay et al., 2020). To assess genetic similarity, ARDRA (Amplified Ribosomal DNA Restriction Analysis) banding patterns are analyzed using Jaccard’s coefficient. The resulting similarity coefficient matrix is then processed using the Unweighted Pair Group Method with Arithmetic Mean (UPGMA) algorithm to generate clusters. This analysis is performed using the NTYSYS 2.02 PC program.

**FIGURE 3:**
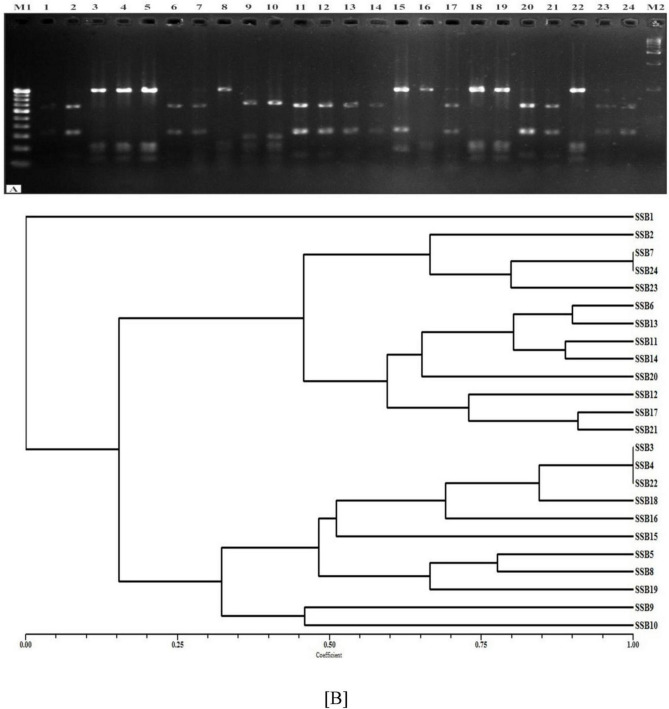
**(A)** Amplification profile based on ARDRA patterns of 24 SiS-RB isolates using restriction endonuclease *Hin*fI. **(B)** Combined dendrogram of 24 SiS-RB isolates based on average similarity coefficients for *Alu*I, *Hae*III, *Taq*I, and *Hin*fI enzyme using the unweighted pair group method with Arithmetic averages (UPGMA).

Four restriction endonucleases viz. *Alu*I*, Hin*fI*, Hae*III, and *Taq*I were used for 16S rDNA RFLP analysis and the banding patterns of the representative SiS-RB with standard molecular weight marker are shown in (Figure 3). In total, 24 bands of varying sizes were observed in all the SiS-RB strains with catalysis by four restriction endonucleases. The *Hin*fI produced 05, *Hae*III produced 08, *Alu*I produced 05, and *Taq*I produced 06 polymorphic bands upon digestion. Jaccard’s similarity coefficient-based banding pattern was used for cluster analysis to study genetic relationship. Similarity indices established on the basis 24 bands of 4 restriction enzymes ranged from 0.11 to 0.90 with an average value of 0.51. The pair wise comparison of ARDRA patterns based on both shared and unique amplification products was made to generate a similarity matrix. The dendrogram is a close representation of the values obtained in the Jaccard similarity matrix discriminated all SSB isolates into two major clusters at 0.15 similarity coefficient. The ARDRA revealed moderate molecular diversity among SSB strains studied in the present study.

The I*^st^* (A) cluster consists of twelve strains and it was divided into sub cluster A1 and A2. Sub cluster A1 included total of four strains *viz.*, SSB2, SSB 7, SSB 23 and SSB 24 Sub cluster A2 included four strains and comprises of eight strains *viz.*, SSB 6, SSB 13, SSB 11, SSB 14, SSB 20, SSB 12, SSB 17, and SSB 21. The second cluster (B) included total eleven strains and it was divided into sub cluster B1 and B2. Sub cluster B1 included total of nine strains *viz.*, SSB3, SSB 4, SSB 22, SSB 18, SSB 16, SSB 15, SSB 5, SSB 8 and SSB 19 Sub cluster B2 included two strains *viz.*, SSB 9 and SSB 10. SSB1 was not grouped in any cluster and was kept as independent strain in the dendrogram (Figure 3). The combined dendrogram, generated using UPGMA based on average similarity coefficients, showed similarity indices ranging from 0.11 to 0.90, with an average of 0.51. This dendrogram closely reflects the Jaccard similarity matrix, which classified all SiS-RB isolates into two major clusters at a similarity coefficient of 0.15, comprising 12 and 11 strains, respectively.

**FIGURE 4:**
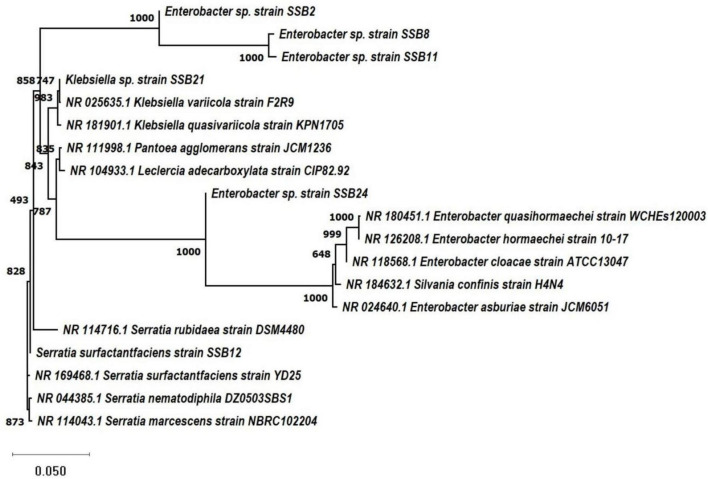
Phylogenetic tree constructed by using submitted sequences of potent SSB2, T2 = SSB8, T3 = SSB11, T4 = SSB12, T5 = SSB21, and T6 = SSB24 isolates with retrieved sequences from GenBank of closely submitted sequences of different species.

In this study, SSB-2 was identified as *Enterobacter sp.* (PQ157604), SSB-8 *was Enterobacter sp.* (PQ157605), SSB-11 was *Enterobacter sp. (*PQ157606), SSB-12 was *Serratia surfactantfaciens (*MW308551), SSB-21 was *Klebsiella sp.* (PQ157607), and SSB-24 was *Enterobacter sp.* (PQ157608). Based on submitted sequences of 16S rDNA of six SiS-RB were used to construct phylogenetic tree (Figure 4), which raveled the close relationship between silica solubilizing microbes. Chandrakala et al. (2019) utilized 16S rDNA gene sequencing to identify the silicate solubilizing IIRI-1 isolate as *Rhizobium sp.* Well-known root nodulation bacteria. Similarly, Raturi et al. (2022) performed comprehensive genome sequencing of *Enterobacter* sp. LR6 of Gram-negative *Enterobacteriaceae* family which also represents the potent isolates of the present study and reported that this family provided a landscape of highlighting genes responsible for silicate solubilization, stress tolerance, and growth-promoting activity.

## 4 Conclusion

This study underscores the considerable potential of rhizospheric bacteria in solubilizing silicate minerals and enhancing plant development. The discovery and characterization of silica-solubilizing plant growth-promoting rhizobacteria (SiS-PGPR), comprising distinct isolates such as *Enterobacter* sp., *Serratia surfactantfaciens*, and *Klebsiella* sp., provide significant insights into sustainable agriculture techniques. These bacteria exhibited significant capacities, including the enhancement of silica solubilization, the promotion of plant growth, and the increase of silica absorption in maize plants. 24 rhizobacterial isolates had robust silica-solubilizing and plant growth-promoting characteristics, while six isolates exhibiting outstanding efficacy in mineral solubilization, plant growth promotion, and enhancement of antioxidant enzyme activities in maize. Further, the dedicated field studies for these SiS-PGPR need to conducted in order to assess its efficacy and mechanism under different soil ecosystems including silicon deficiency conditions. Utilizing the capabilities of these effective SiS-PGPR for the development of effective biofertilizer formulations, agricultural systems may get improved crop yield and silica management while decreasing dependence on chemical fertilizers. This research underscores the significance of biofertilizers in advancing sustainable agriculture while minimizing environmental effect, in accordance with the global sustainable development goal.

## Data Availability

The datasets presented in this study can be found in online repositories. The names of the repository/repositories and accession number(s) can be found below: https://www.ncbi.nlm.nih.gov/genbank/, PQ157604, PQ157605, PQ157606, MW308551. PQ157607, PQ157608.
