# Correction: Bioprospecting of novel silica solubilizing bacteria as bioinoculants for sustainable silica management

**DOI:** 10.3389/fmicb.2025.1717737

**Published:** 2025-10-22

**Authors:** Elina Maharjan, Sonam Mahawar, Surya Chauhan, Sudhir Kumar Upadhyay, Santosh Ranjan Mohanty, Ajaz Ahmad, Rajesh Kumar Singh, Devendra Jain

**Affiliations:** ^1^Central Department of Microbiology, Tribhuvan University, Kirtipur, Nepal, India; ^2^All India Network Project on Soil Biodiversity- Biofertilizers, Department of Molecular Biology and Biotechnology, Maharana Pratap University of Agriculture and Technology, Udaipur, India; ^3^Research and Development Cell, Lovely Professional University, Phagwara, Punjab, India; ^4^Indian Institute of Soil Science, Indian Council of Agricultural Research, Bhopal, Madhya Pradesh, India; ^5^Department of Clinical Pharmacy, College of Pharmacy, King Saud University, Riyadh, Saudi Arabia; ^6^Key Laboratory of Sugarcane Biotechnology and Genetic Improvement (Guangxi), Ministry of Agriculture, Sugarcane Research Center, Chinese Academy of Agricultural Sciences, Nanning, Guangxi, China

**Keywords:** silica solubilizing rhizobacteria, mineralization, phyto-stimulation, antioxidants, ARDRA, 16S rDNA

Affiliation 1, “*Central Department of Microbiology, Tribhuvan University, Kirtipur, Nepal, India*” was erroneously assigned to Devendra Jain. Devendra Jain's correct affiliation is Affiliation 2 “*All India Network Project on Soil Biodiversity- Biofertilizers, Department of Molecular Biology and Biotechnology, Maharana Pratap University of Agriculture and Technology, Udaipur, India”*.

The original version of this article has been updated.

