# Supplementary material for: Bioprospecting of novel silica solubilizing bacteria as bioinoculants for sustainable silica management
Source: Front Microbiol. 2025 Jun 9;16:1556406. doi: 10.3389/fmicb.2025.1556406 (PMC12183168; doi:10.3389/fmicb.2025.1556406)
Supplement: Supplementary file 1 [file Supplementary_file_1.docx]

Supplementary Material

# Supplementary Table 1

**Table 1. Silica solubilizing bacterial isolates with their isolation source and morphological characteristics**

| **S. No.** | **Strains** | **Source** | **GPS Location** | **Gram staining** | **Shape** |
| --- | --- | --- | --- | --- | --- |
| 1 | SSB-1 | Kumbhalgarh | 25.056013, 73.494293 | Gm-ve | Cocci |
| 2 | SSB-2 | Kumbhalgarh | 25.056013, 73.494293 | Gm-ve | Cocci |
| 3 | SSB-3 | Kumbhalgarh | 25.056013, 73.494293 | Gm-ve | Cocci |
| 4 | SSB-4 | Kumbhalgarh | 25.056013, 73.494293 | Gm-ve | Cocci |
| 5 | SSB-5 | Kumbhalgarh | 25.056013, 73.494293 | Gm-ve | Cocci |
| 6 | SSB-6 | Kumbhalgarh | 25.056013, 73.494293 | Gm-ve | Cocci |
| 7 | SSB-7 | Kumbhalgarh | 25.056013, 73.494293 | Gm-ve | Cocci |
| 8 | SSB-8 | RCA | 24.582754, 73.705337 | Gm-ve | Cocci |
| 9 | SSB-9 | RCA | 24.582754, 73.705337 | Gm-ve | Cocci |
| 10 | SSB-10 | RCA | 24.582754, 73.705337 | Gm-ve | Cocci |
| 11 | SSB-11 | RCA | 24.582754, 73.705337 | Gm-ve | Cocci |
| 12 | SSB-12 | RCA | 24.582754, 73.705337 | Gm-ve | Cocci |
| 13 | SSB-13 | RCA | 24.582754, 73.705337 | Gm-ve | Rod |
| 14 | SSB-14 | RCA | 24.582754, 73.705337 | Gm-ve | Rod |
| 15 | SSB-15 | RCA | 24.582754, 73.705337 | Gm-ve | Rod |
| 16 | SSB-16 | RCA | 24.582754, 73.705337 | Gm-ve | Rod |
| 17 | SSB-17 | RCA | 24.582754, 73.705337 | Gm-ve | Rod |
| 18 | SSB-18 | RCA | 24.582754, 73.705337 | Gm+ve | Rod |
| 19 | SSB-19 | RCA | 24.582754, 73.705337 | Gm-ve | Cocci |
| 20 | SSB-20 | RCA | 24.582754, 73.705337 | Gm-ve | Rod |
| 21 | SSB-21 | RCA | 24.582754, 73.705337 | Gm-ve | Rod |
| 22 | SSB-22 | RCA | 24.582754, 73.705337 | Gm-ve | Rod |
| 23 | SSB-23 | RCA | 24.582754, 73.705337 | Gm+ve | Cocci |
| 24 | SSB-24 | RCA | 24.582754, 73.705337 | Gm-ve | Rod |

# Supplementary Table 2

**Supplementary Table 2:** Plant Growth Promoting Activities of SiS-RB isolates

| SiS-RB isolates | IAA | GA_3_ | Phosphorus solubilization | Potassium solubilization | Zinc solubilization | ACC-deaminase activity | Ammonia production | HCN  production |
| --- | --- | --- | --- | --- | --- | --- | --- | --- |
| SSB-1 | 3.12 | 0.485 | 1.05±0.10 | 2.4± 0.2 | 2.57± 0.29 | + | + | + |
| SSB-2 | 1.29 | 0.705 | 2.5± 0.19 | 3± 0.25 | 5± 0.33 | + | + | + |
| SSB-3 | 7.691 | 0.564 | 1.04 ± 0.14 | 2.8± 0.208 | 3.75± 0.27 | + | - | + |
| SSB-4 | 18.32 | 0.543 | 1.04± 0.17 | 3± 0.32 | 2.16± 0.14 | + | + | + |
| SSB-5 | 43.92 | 0.456 | 1.048± 0.08 | 1.42± 0.10 | 1.5± 0.169 | + | - | - |
| SSB-6 | 15.97 | 0.523 | 1.03± 0.19 | 1.36± 0.16 | 3.6± 0.23 | + | - | - |
| SSB-7 | 10.26 | 0.417 | 1.15± 0.11 | 2± 0.17 | 2.42± 0.27 | - | + | - |
| SSB-8 | 8.54 | 0.645 | 1.6± 0.198 | 3.5± 0.26 | 4.2± 0.10 | ++ | - | - |
| SSB-9 | 15.74 | 0.385 | 1.01±0.28 | 2± 0.18 | 1.85± 0.18 | - | + | - |
| SSB-10 | 12.03 | 0.432 | 1.09± 0.32 | 1.25± 0.05 | 2.6± 0.13 | + | - | + |
| SSB-11 | 14.83 | 0.617 | 1.6±0.18 | 3.4± 0.20 | 4.25± 0.22 | + | - | - |
| SSB-12 | 4.32 | 0.652 | 1.16± 0.22 | 3± 0.179 | 5± 0.21 | + | + | + |
| SSB-13 | 11.17 | 0.509 | 1.01± 0.22 | 2± 0.22 | 2.57± 0.30 | + | - | + |
| SSB-14 | 17.63 | 0.489 | 1.01± 0.35 | 2.5± 0.091 | 2.85± 0.28 | - | + | + |
| SSB-15 | 4.83 | 0.417 | ND | ND | 2.6± 0.18 | + | + | - |
| SSB-16 | 18.14 | 0.419 | 1.07± 0.28 | 1.2± 0.058 | 3.5± 0.15 | + | + | - |
| SSB-17 | 18.20 | 0.393 | 1.6± 0.164 | 2.8± 0.165 | 3.8± 0.22 | + | - | + |
| SSB-18 | 12.66 | 0.543 | ND | ND | 1.75± 0.21 | - | + | + |
| SSB-19 | 6.60 | 0.517 | 1.028± 0.20 | 1.25± 0.05 | 1.83± 0.31 | + | + | - |
| SSB-20 | 15.63 | 0.568 | 1.011± 0.17 | 1.42± 0.15 | 1.42± 0.12 | + | - | + |
| SSB-21 | 5.69 | 0.605 | 1.75± 0.21 | 3.25± 0.12 | 3.75± 0.27 | + | + | + |
| SSB-22 | 4.66 | 0.378 | 1.06± 0.03 | 2.6± 0.172 | 1.85± 0.13 | - | - | - |
| SSB-23 | 5.97 | 0.387 | 1.01± 0.214 | 1.36±0.152 | 1.8± 0.18 | + | - | - |
| SSB-24 | 4.32 | 0.711 | 3.0± 0.21 | 6± 0.27 | 4.25± 0.29 | + | + | + |

ND=Not detected

# Supplementary Table 3

**Supplementary Table 3:** Different Biochemical characterization of SiS-RB isolates

| SiS-RB isolates | Starch Hydrolysis | Citrate Utilization | Nitrate Reduction | Gelatin liquefaction | Catalase Activity | Oxidase |
| --- | --- | --- | --- | --- | --- | --- |
| SSB-1 | **+** | **+** | **-** | **-** | **+** | **-** |
| SSB-2 | **+** | **+** | **-** | **-** | **+** | **-** |
| SSB-3 | **+** | **+** | **+** | **-** | **+** | **-** |
| SSB-4 | **-** | **+** | **+** | **+** | **+** | **-** |
| SSB-5 | **-** | **+** | **+** | **-** | **+** | **-** |
| SSB-6 | **-** | **+** | **-** | **-** | **+** | **-** |
| SSB-7 | **+** | **+** | **-** | **-** | **+** | **-** |
| SSB-8 | **+** | **+** | **+** | **-** | **+** | **+** |
| SSB-9 | **+** | **+** | **+** | **-** | **+** | **-** |
| SSB-10 | **+** | **+** | **+** | **-** | **+** | **-** |
| SSB-11 | **+** | **+** | **+** | **-** | **+** | **-** |
| SSB-12 | **+** | **+** | **+** | **+** | **+** | **+** |
| SSB-13 | **-** | **+** | **+** | **-** | **+** | **-** |
| SSB-14 | **-** | **+** | **+** | **+** | **+** | **-** |
| SSB-15 | **+** | **-** | **+** | **+** | **+** | **-** |
| SSB-16 | **+** | **+** | **+** | **-** | **+** | **-** |
| SSB-17 | **+** | **+** | **-** | **-** | **+** | **-** |
| SSB-18 | **+** | **+** | **+** | **-** | **+** | **-** |
| SSB-19 | **-** | **+** | **+** | **-** | **-** | **-** |
| SSB-20 | **-** | **+** | **+** | **-** | **+** | **+** |
| SSB-21 | **-** | **+** | **+** | **-** | **+** | **-** |
| SSB-22 | **-** | **+** | **+** | **-** | **+** | **+** |
| SSB-23 | **-** | **-** | **-** | **-** | **+** | **-** |
| SSB-24 | **+** | **+** | **+** | **-** | **+** | **-** |
